# Blood lead levels in Peruvian adults are associated with proximity to mining and DNA methylation

**DOI:** 10.1016/j.envint.2021.106587

**Published:** 2021-04-30

**Authors:** Ainash Childebayeva, Jaclyn M. Goodrich, Nathan Chesterman, Fabiola Leon-Velarde, Maria Rivera-Ch, Melisa Kiyamu, Tom D. Brutsaert, Abigail W. Bigham, Dana C. Dolinoy

**Affiliations:** a Department of Anthropology, University of Michigan, Ann Arbor, MI 48109, USA; b Department of Environmental Health Sciences, School of Public Health, University of Michigan, Ann Arbor, MI 48109, USA; c Department of Archaeogenetics, Max Planck Institute for the Science of Human History, Jena 07745, Germany; d School for Environment and Sustainability, University of Michigan, Ann Arbor, MI 48109, USA; e Departamento de Ciencias Biológicas y Fisiológicas, Facultad de Ciencias y Filosofía, Universidad Peruana Cayetano Heredia, Lima, Peru; f Department of Exercise Science, Syracuse University, Syracuse, NY 13244, USA; g Department of Anthropology, University of California, Los Angeles, CA 90095, USA; h Department of Nutritional Sciences, School of Public Health, University of Michigan, Ann Arbor, MI 48109, USA

**Keywords:** Lead (Pb), Hemoglobin, EWAS, Andes, Mining, DNA methylation

## Abstract

**Background:**

Inorganic lead (Pb) is common in the environment, and is toxic to neurological, renal, and cardiovascular systems. Pb exposure influences the epigenome with documented effects on DNA methylation (DNAm). We assessed the impact of low levels of Pb exposure on DNAm among non-miner individuals from two locations in Peru: Lima, the capital, and Cerro de Pasco, a highland mining town, to study the effects of Pb exposure on physiological outcomes and DNAm.

**Methods:**

Pb levels were measured in whole blood (n = 305). Blood leukocyte DNAm was determined for 90 DNA samples using the Illumina MethylationEPIC chip. An epigenome-wide association study was performed to assess the relationship between Pb and DNAm.

**Results:**

Individuals from Cerro de Pasco had higher Pb than individuals from Lima (p-value = 2.00E-16). Males had higher Pb than females (p-value = 2.36E-04). Pb was positively associated with hemoglobin (p-value = 8.60E-04). In Cerro de Pasco, blood Pb decreased with the distance from the mine (p-value = 0.04), and association with soil Pb was approaching significance (p-value = 0.08). We identified differentially methylated positions (DMPs) associated with genes *SOX18, ZMIZ1,* and *KDM1A* linked to neurological function. We also found 45 differentially methylated regions (DMRs), seven of which were associated with genes involved in metal ion binding and nine to neurological function and development.

**Conclusions:**

Our results demonstrate that even low levels of Pb can have a significant impact on the body including changes to DNAm. We report associations between Pb and hemoglobin, Pb and distance from mining, and between blood and soil Pb. We also report associations between loci- and region-specific DNAm and Pb.

## 1. Introduction

Lead (Pb) is a trace metal that has been associated with human civilization since the emergence of metallurgy (Mielke and Reagan 1998). Pb is the world’s most abundant heavy metal, and exposure to it is estimated to account for 143,000 deaths per year, accounting for 0.6% of the global disease burden (Haefliger 2011; Tong et al. 2000). Miners and mining communities are at a higher risk of exposure to Pb and its effects, including higher levels of Pb in both indoor and outdoor air, soil, and self-produced vegetables in areas close to Pb mining (Attina and Trasande 2013; Qu et al. 2012).

Pb exposure can have negative effects on any organ in the body, including the neurological, hematological, cardiovascular, and renal systems, with the nervous system being the most susceptible to its harmful effects (Kalia and Flora 2005; Sanders et al. 2009; WHO 2011). Both central and peripheral nervous systems can be affected by Pb (Flora et al. 2012). In children, Pb exposure has been associated with encephalopathy characterized by a degeneration of certain parts of the brain (Sanders et al. 2009). Moreover, children exposed to Pb may experience delayed growth, decreased IQ, impairment in concentration ability, and other adverse outcomes (Flora et al. 2012; Sanders et al. 2009). Additionally, Pb has a direct effect on the hematopoietic system by inhibiting enzymes involved in the heme synthesis pathway, thus leading to decreased hemoglobin (Hb) concentration (Barltrop and Smith 1971; Hu et al. 1994). Pb also increases the fragility of erythrocyte membranes, reducing their lifespan and resulting in anemia (Flora et al. 2012).

There is growing evidence that Pb can impact the epigenome. Epigenetic modifications can have an effect on gene expression without changing the sequence of the nucleotides. DNA methylation (DNAm) is the most commonly studied epigenetic modification and is generally associated with gene repression when located in promoter regions of genes (Fraga and Esteller 2002). Inorganic Pb may affect DNAm via increased production of reactive oxygen species (ROS) upon exposure (Adonaylo and Oteiza 1999). Increased oxidative stress is known to result in decreased DNAm (Pogribny et al. 2007), and can inhibit binding of the methyl-CpG binding proteins that promote chromatin inactivation (Valinluck et al. 2004).

Previous studies have shown associations between Pb exposure and both global, gene-specific, and genome-wide DNAm signatures in humans and human embryonic stem cells (Goodrich et al. 2016; Hanna et al. 2012; Li et al. 2013; Li et al. 2016; Rygiel et al. 2020; Senut et al. 2014; Wright et al. 2010). However, little is known about the effects of chronic low-level Pb exposure on genome-wide DNAm in adults exposed to mining who are not miners themselves. This study was performed in a cohort of individuals who were recruited in Lima (low altitude) and Cerro de Pasco, Peru (high altitude) to study genetic and epigenetic aspects of high-altitude adaptation (Brutsaert et al. 2019; Childebayeva et al. 2019). All of the high-altitude participants were residents of Cerro de Pasco, Peru. This municipality is an important Peruvian mining center that was established in the 17th century to support silver mining, and was at one point the second largest producer of silver in the Andes (Fisher 1977; Helfgott 2013). Upon the depletion of the city’s silver reserves, silver mining was replaced by copper, zinc, and Pb extraction (van Geen et al. 2012). In 1952, the Cerro de Pasco Corporation claimed to be the largest South American producer of refined Pb (Helfgott 2013). Given the effect of Pb on the epigenome (Bakulski et al. 2012; Faulk et al. 2014; Goodrich et al. 2015), we measured whole-blood lead levels (WBLLs) in a subset of the study participants (Childebayeva et al. 2019). Here, we performed an epigenome-wide association study to identify DNAm differences between Pb exposure groups. We also determined the relationship between Pb and Hb, proximity to mining, and soil Pb

## 2. Materials and Methods

### 2.1. Study population

**Fig. 1.**
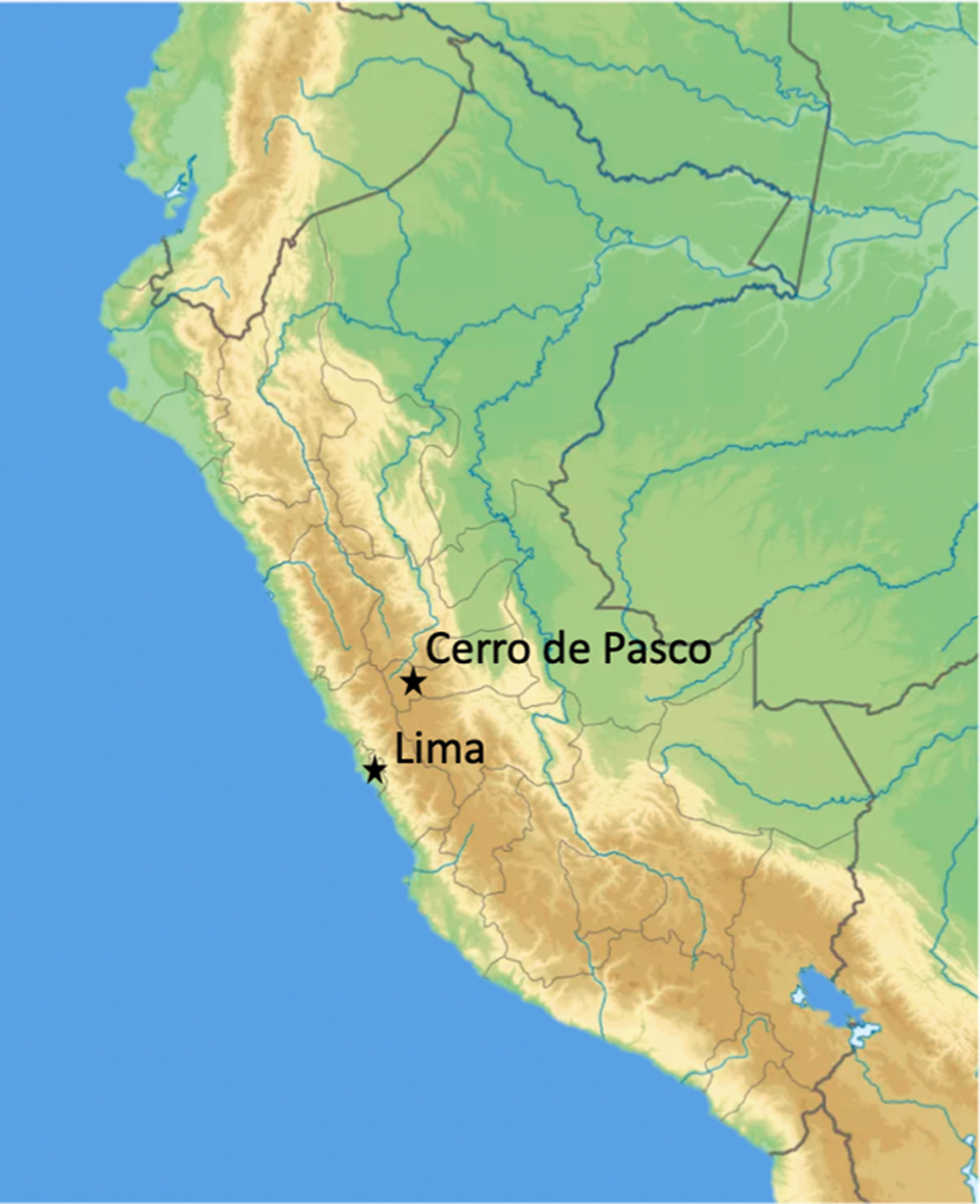
Study Design. Map of Peru depicting high-altitude, Cerro de Pasco (4,338 m), and low-altitude, Lima (150 m), participant recruitment locations.

Individuals in this study (N = 305) were selected from a larger cohort of 603 Peruvian individuals of self-reported multigenerational Quechua ancestry between ages 18 and 35 (Childebayeva et al. 2019). The individuals were recruited based on a migrant study design to study high-altitude adaptation in two locations, Lima and Cerro de Pasco, Peru: Lima is the capital of Peru located at low altitude and the largest city, while Cerro de Pasco is a mining community located at approximately 4,400 m above sea level (masl) (Fig. 1). We determined WBLLs in a subset of the participants (N = 305) (Table 1): 1) Cerro de Pasco (n = 157): Quechua individuals who were born and raised at high altitude and recruited in Cerro de Pasco, a Peruvian mining center; 2) Lima (n = 148): Quechua individuals living in Lima at low altitude as young adults at the time of study, some of whom were born and raised in Lima and some of whom were born in various high-altitude locations in Peru and moved to Lima prior to recruitment. Male and female participants were recruited to participate if they were unrelated, healthy, non-pregnant/lactating, non-smokers, non-miners, and between 18 and 35 years old (Table 1). At the time of enrollment, all study participants provided written informed consent approved by Syracuse University, Universidad Peruana Cayetano Heredia, and the University of Michigan Institutional Review Boards.

### 2.2. Phenotypic assessment and Pb exposure assessment

Height, weight, and education information were collected at the time of enrollment, and the participants provided a venous blood sample into a collection tube containing EDTA (Lavender-top, BD Vacutainer, Franklin Lakes, NJ) for DNA extraction. The Puregene DNA purification system (Qiagen, Valencia, CA) was used to extract the genomic DNA according to the manufacturer’s instructions. Red blood cell lysis was performed in Peru immediately following venous puncture. Samples were stabilized at room temperature in cell lysis solution and transported to the University of Michigan for DNA extraction. Hb levels were determined in venous blood using the HemoCue 201 + analyzer (Angelholm Sweden). All study participants were screened for anemia using altitude specific cut-offs. WBLLs were measured using graphite furnace atomic absorption spectrometry (GFAAS) (Parsons and Slavin 1993). GFAAS measurement was performed in duplicate at the Blufstein Laboratory located in Lima, Peru (coefficient of variance between the duplicates for the entire cohort < 2%).

### 2.3. Geocoding and soil Pb interpolation

Geographic coordinates for the participants from Cerro de Pasco were determined using a portable Garmin GPS (Garmin, Olathe, Kansas) based on address information received during recruitment. We determined the Euclidean distance from the mine (mine-distance) to each participant’s current place of residence using the function distm (function Harvesine) in the R package geosphere (Hijmans et al. 2017).

A previous study by Van Geen et al. (2012) recorded soil Pb values at 74 locations throughout Cerro de Pasco at varying distances from the mine in May 2009, using a hand-held X-ray fluorescence analyzer (Innov-X Alpha, Olympus Corporation, Tokyo, Japan) (van Geen et al. 2012). Using the Pb values reported by van Geen et al. (2012), we interpolated soil Pb levels across Cerro de Pasco using ordinary kriging (Graler et al. 2016). Kriging is a geostatistical interpolation method that produces a prediction surface based on points with known values, and incorporates a semivariogram model to account for the estimated spatial autocorrelation between these points (Burgess and Webster 1980; Krige 1966). We created the predicted surface of soil Pb values using ordinary kriging in ArcGIS 10.4, with a spherical semivariogram model and a cell size of 30 m. In addition to a prediction surface, kriging also creates a surface of estimated prediction error based on the distance between points. This approach has been previously used to interpolate blood Pb level risk with Pb in water for children in Flint, MI (Hanna-Attisha et al. 2016). We used the estimated soil Pb across Cerro de Pasco to estimate soil Pb levels at each participating household in Cerro de Pasco.

### 2.4. DNA methylation

A random subset of participants was selected from both Lima (n = 59) and Cerro de Pasco (n = 31) for DNAm analysis. EZ-96 DNA Methylation^™^ Kit (Zymo Research, Irvine, CA) was used for bisulfite conversions of all DNA samples following the standard protocol with alternative incubation conditions optimized for the Illumina Infinium^®^ MethylationEPIC BeadChip (Zymo, Irvine, California). Raw methylation intensity data was loaded into R using the package minfi (Aryee et al. 2014). Eighty-seven samples passed QC, and their missing beta values were imputed, normalized using functional normalization (FunNorm) in minfi followed by the beta-mixture quantile normalization (BMIQ) (Fortin et al. 2014; Morris et al. 2014; Teschendorff et al. 2013). We removed probes with detection p-values > 10e-5 in>10% of samples, probes identified as cross-reactive, probes containing common SNPs, and probes on X and Y chromosomes (for more information on the pipeline see Sup. Fig. 2) (Chen et al. 2013; Pidsley et al. 2016). The resulting beta values (n = 656,183) were tested for batch effects using Singular Value Decomposition (SVD) (Sup. Fig. 3), and the batch effects were corrected using Combat in ChAMP (Johnson et al. 2007; Teschendorff et al. 2009). Following batch correction, beta values were converted into M—values, and differentially methylated positions (DMPs) were determined using limma (Ritchie et al. 2015).

Differentially methylated regions (DMRs) were identified based on the uncorrected p-values (p < 0.05) from the DMP limma analysis using comb-p with the following options (comb-p pipeline -c 4 –seed 10e-3–dist 3000). DMRs were corrected for multiple comparisons using the built-in Sidak correction function in comb-p (Pedersen et al. 2012). Pathway analysis was performed on significant sites (unadjusted p-value < 0.05) from the limma analysis of DMPs using Consensus PathDB (Herwig et al. 2016; Kamburov et al. 2011). We used DAVID for functional annotation of the DMRs (Dennis et al. 2003) using the annotations from Gene Ontology (Ashburner et al. 2000; Gene Ontology 2021), UniProt KrowledgeBase (UniProtKB) (UniProt 2019), BIOCARTA (Nishimura 2001), and Kyoto Encyclopedia of Genes and Genomes (KEGG) (Wixon and Kell 2000).

### 2.5. Statistical analyses

All statistical analyses on WBLLs and phenotypes of interest were conducted using R version 3.4.0 (R Core Team, 2020). Package ggplot2 (Wickham 2009) was used to plot the data. We ran linear models to assess the relationship between interpolated soil Pb values and WBLLs in a subset of participants from Cerro de Pasco whose geographical location we were able to determine based on their residence location (n = 152). We used interpolated soil Pb values for each participant in the following regression model to find associations between soil Pb and WBLLs: Yi (WBLLs) ~ B00 + B01(interpolated soil Pb) + B02(Sex) + B03(Education) + B04(Kriging standard error), where Yi is the dependent variable, WBLL; “interpolated soil Pb” = interpolated soil Pb value.

DMPs were determined in limma using the following model: DNAm ~ WBLLs + Group (i.e. Altitude) + Sex + Age + Blood principal components (PCs) 1,2,5,6. Blood PCs were determined based on the estimated cell counts function proposed by Houseman et al. (2012). Benjamini-Hochberg correction was used to control for multiple testing and a false discovery rate of 10% was considered significant (Ritchie et al. 2015). Distribution of expected and observed p-values were used to validate the model fit (Sup. Fig. 4).

The relationship between WBLLs and hemoglobin was tested using the following model: Hb ~ WBLLs + Group (i.e. Altitude) + sex.

## 3. Results

### 3.1. Study design and participant characteristics

Participant characteristics are provided in Table 1. The study groups did not differ significantly by sex or age. Individuals recruited in Cerro de Pasco were significantly shorter, lighter, and had lower BMI than individuals recruited in Lima (Table 1).

### 3.2. Lead levels are associated with study group, sex, and Hb

**Fig. 2.**
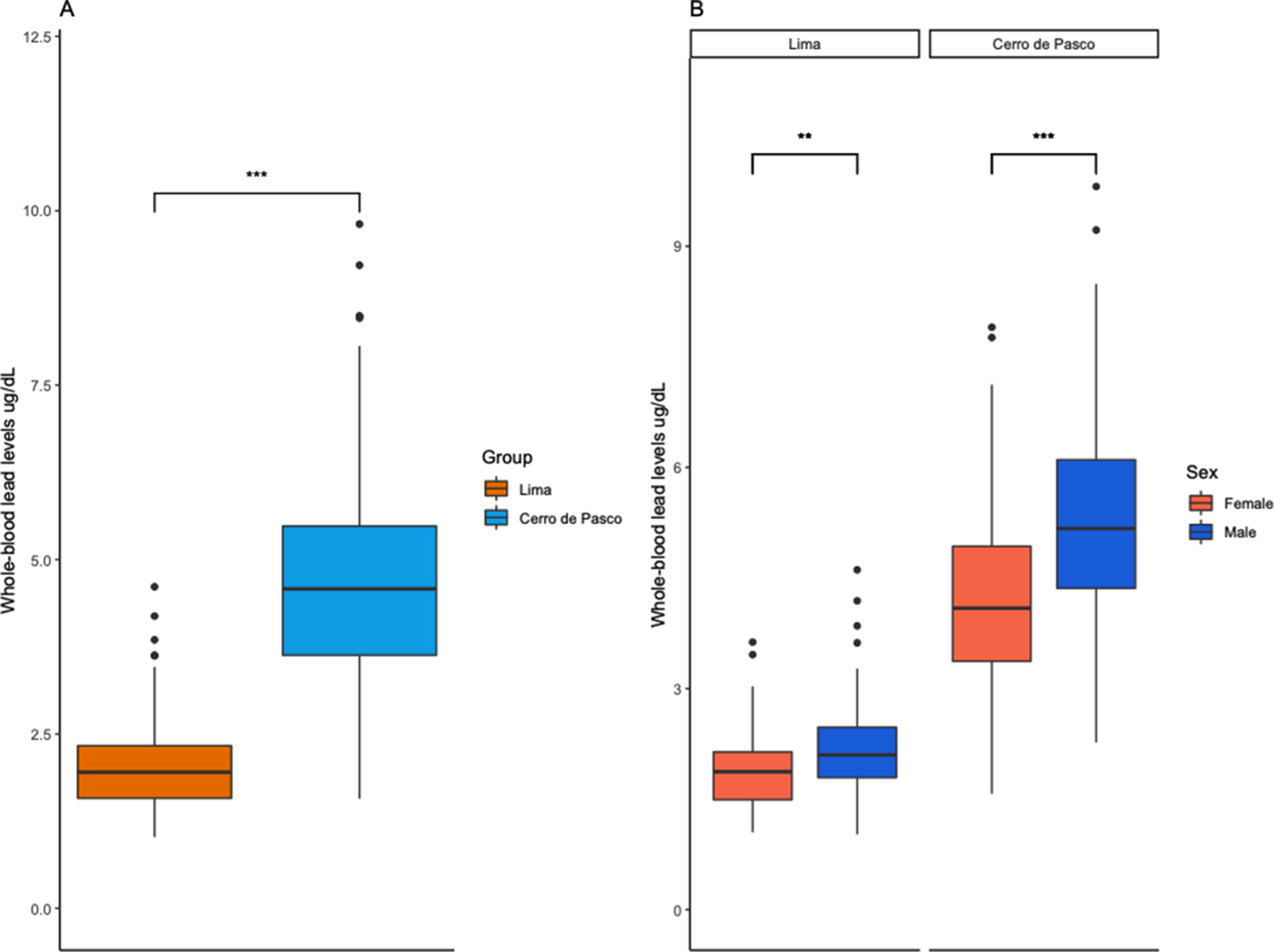
Whole-blood lead levels (WBLLs) in Lima and Cerro de Pasco. (A) Participants from Cerro de Pasco have higher WBLLs than the participants from Lima (p-value < 2.2e-16). (B) WBLLs between Cerro de Pasco and Lima by sex. Males have higher WBLLs than females in both cities (Lima: p-value = 0.0024; Cerro de Pasco: p-value = 1.13E-06).

WBLLs were associated with study group (Fig. 2A, Table 1). Participants from Cerro de Pasco had significantly higher WBLLs (4.75 ± 1.53 μg/dL) compared to the participants recruited in Lima (2.03 ± 0.62 μg/dL) based on linear modeling (β = 2.72, p-value = 2e-16). Sex was significantly associated with WBLLs in both Lima and Cerro de Pasco, with males having higher Pb levels than females (Fig. 2B) in each group (Lima: β = 0.31, p-value = 2.40E-03, Cerro de Pasco: β = 1.15, p-value = 1.13e-06).

**Fig. 3.**
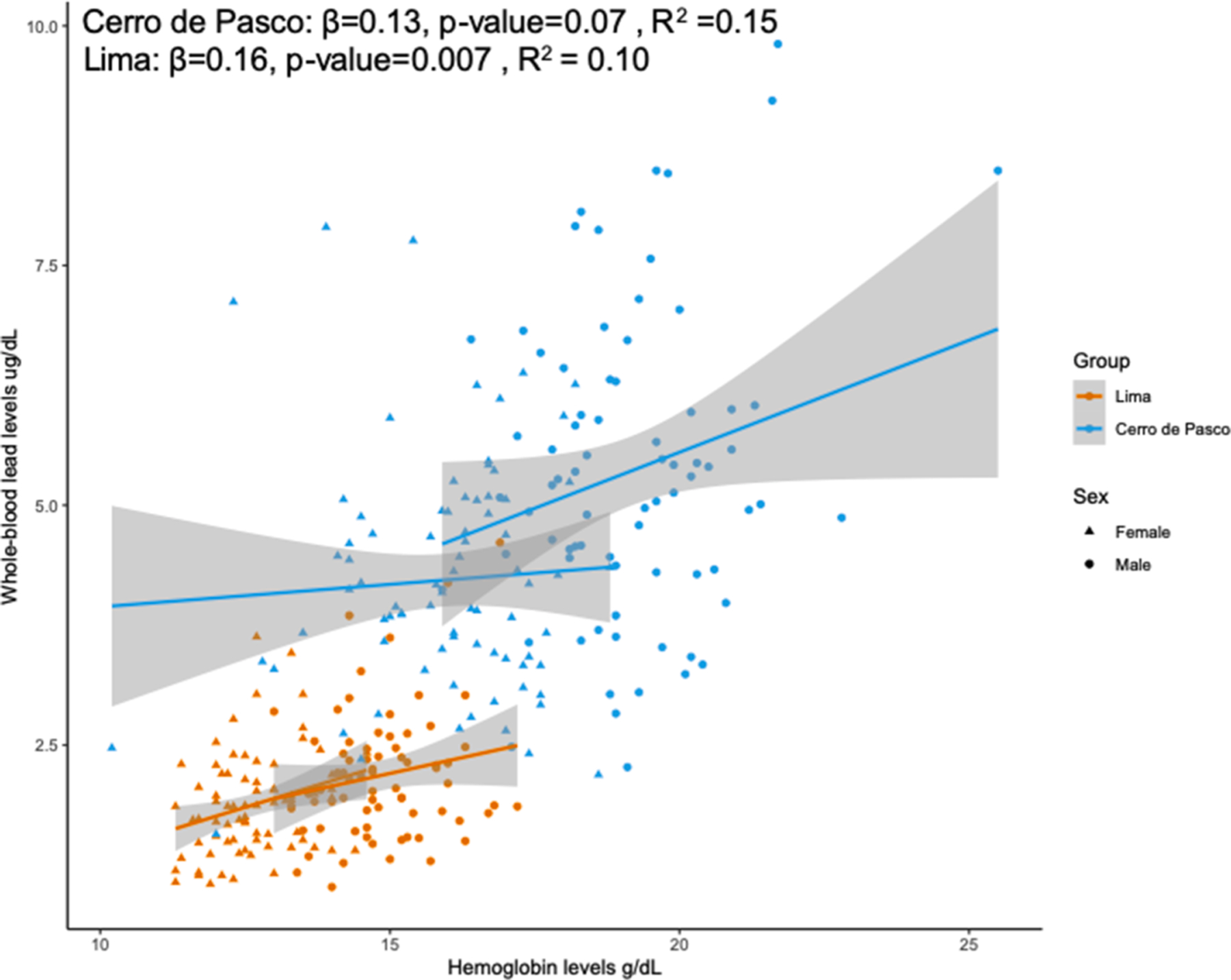
Association between hemoglobin (Hb) in participants from Lima and Cerro de Pasco. The association between WBLLs and Hb levels was not significant for individuals from Cerro de Pasco. The association was significant for individuals in Lima. Statistical models were adjusted for sex.

We identified a positive association between WBLLs and Hb concentration when adjusted for sex and sample group (i.e. altitude) in the entire cohort (β = 0.17, p-value = 8.60E-04) (Fig. 3, Sup. Table 1). When considering the groups separately, WBLLs were significantly associated with Hb levels in participants from Lima (β = 0.32, p-value = 0.007), but not in Cerro de Pasco; although the model was approaching significance (β = 0.16, p-value = 0.07).

### 3.3. Blood lead is negatively correlated with distance from the mine and positively correlated with soil lead in Cerro de Pasco

**Fig. 4.**
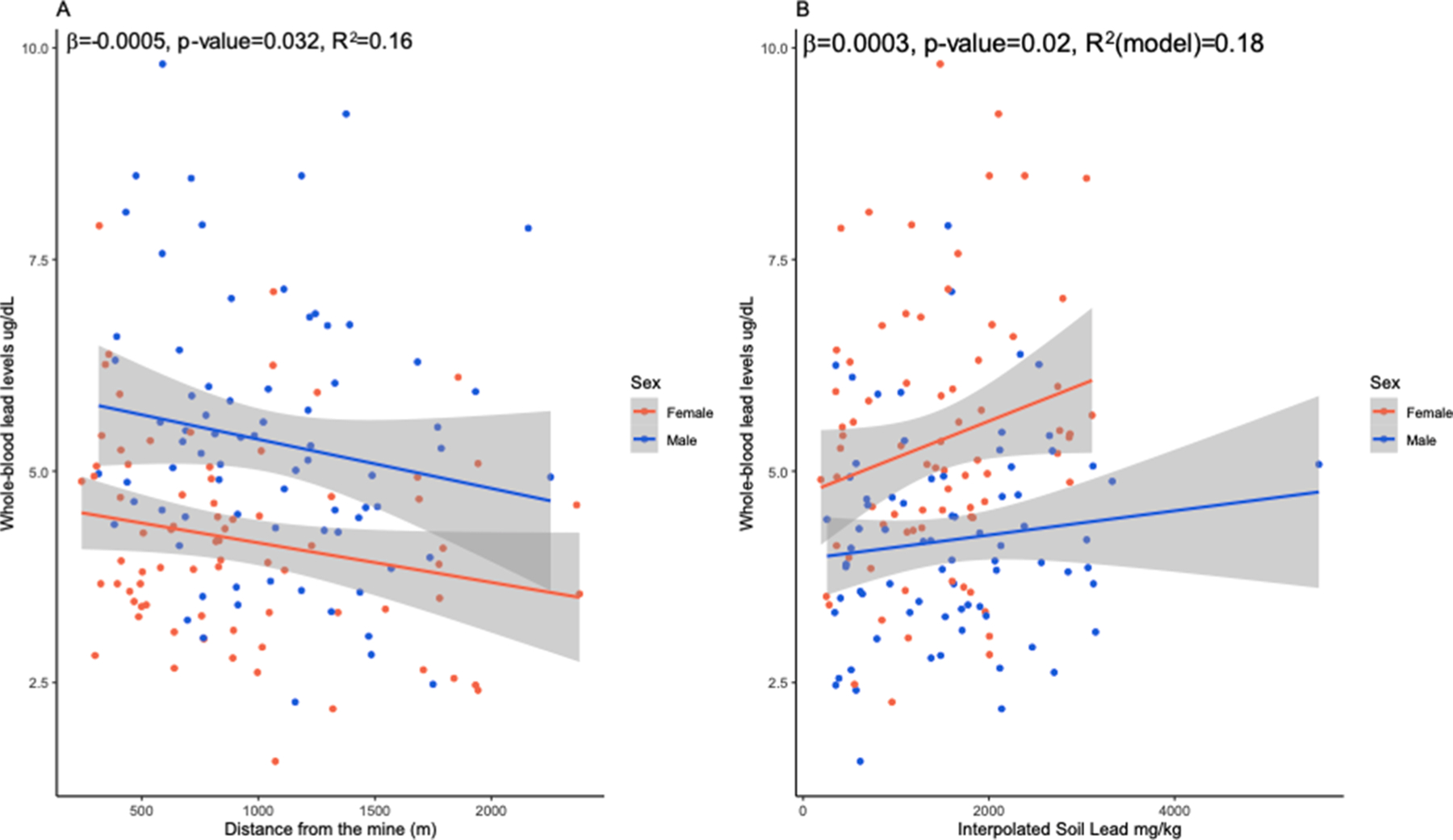
Whole-blood lead levels plotted against the distance from the mine, and interpolated soil lead values. (A) Whole-blood lead levels (WBLL) were negatively associated with distance from the mine in the participants from Cerro de Pasco (model adjusted for age and sex). Unadjusted WBLLs are plotted against the distance from the mine. (B) Relationship between interpolated soil Pb and blood Pb in participants from Cerro de Pasco. The statistical model testing for this association was adjusted for sex and education level. Unadjusted WBLLs are plotted against the interpolated soil Pb.

**Fig. 5.**
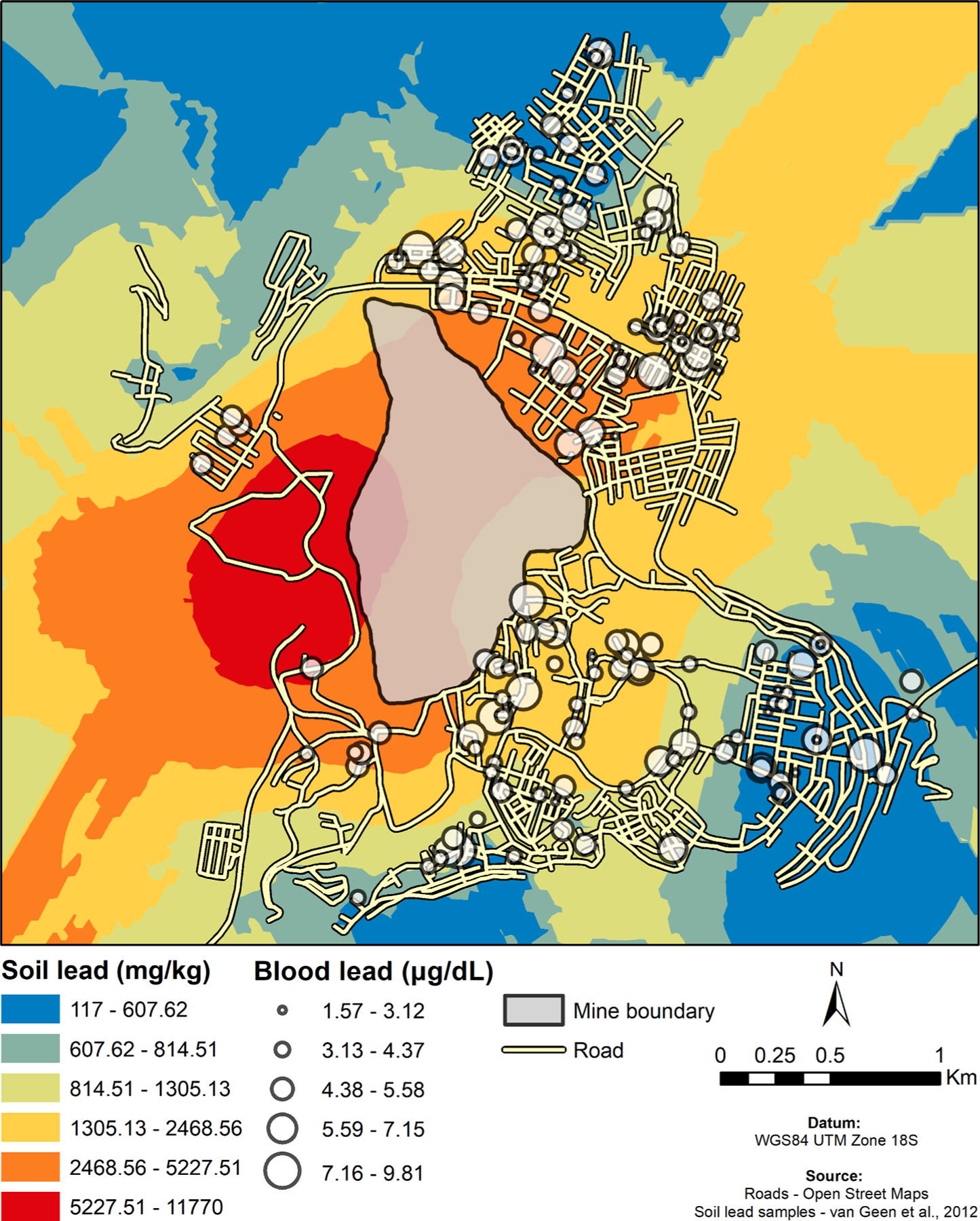
Interpolated soil Pb values for participants from Cerro de Pasco. Soil Pb values were interpolated for participants from Cerro de Pasco using the kriging option in ArcGIS.

The distance from the mine was negatively associated with WBLLs when adjusted for sex and education (Fig. 4A, β = 0.0005, p-value = 0.032). Association between WBLLs and interpolated soil Pb (mg/kg) values, adjusted for sex, education, and Kriging standard error, was approaching significance (β = 0.0003, p-value = 0.08) (Sup. Table 1, Fig. 4B and Fig. 5).

### 3.4. DNA methylation and blood lead

We performed a singular value decomposition (SVD) analysis of genome-wide DNAm data to capture the most salient factors of variation in the data with a small number of components (Teschendorff et al., 2009). Based on SVD, WBLLs were among the significant components of DNAm variation (p < 0.05) before and after experimental batch effects adjustment (Sup. Fig. 3).

We identified four significant DMPs, i.e. single CpG sites, associated with WBLLs at q-value < 0.10 in a model adjusted for study group, sex, age, and blood principal components; the genome-wide inflation factor for this model was 1.01 (Table 2). These DMPs were within and/or in regulatory regions of genes *KDM1A*, *ZMIZ1, TERT, and SOX18* (Sup. Fig. 1). We performed a pathway analysis of the probes significant at the unadjusted p-value < 0.05 over all probes as background using ConsensusPathDB. Based on which, we found n = 22 enriched gene ontology-based sets under the q-value 0.05 cutoff with “Neuronal System” (Reactome pathway, p-value = 4.03e-07, q-value = 0.00154) as the top hit (Sup. Table 2).

DMRs reflect several CpG sites in close proximity changing in the same direction. Therefore, they are considered more representative of biologically relevant epigenetic changes than single methylation sites (i. e. DMPs). We identified 45 significant DMRs, i.e. regions with three or more significant DMPs within a 3000 base-pair window, using comb-p (Sup. Table 3). Out of the significant DMRs, seven were associated with metal ion binding and nine with neurological function and development based on an annotation from DAVID (Dennis et al. 2003) (Table 3).

## 4. Discussion

Pb exposure and the resulting Pb toxicity can negatively affect almost any system of the body, and thus represent an important environmental health and safety concern. Our results identified differences in DNAm, mainly decreased methylation, in Peruvian adults with higher levels of Pb exposure. These differentially methylated positions and regions were associated with genes involved in neurological development and function as well as metal ion binding. Additionally, Pb was positively associated with Hb concentration and soil Pb and inversely associated with proximity to mining. Collectively, our study contributes to the body of knowledge on Pb toxicity in a cross-sectional study of healthy adults who are not miners exposed to Pb from a residential mine.

We identified DNAm differences, both at the level of individual CpG sites (DMPs) and in regions of multiple neighboring CpG sites (DMRs), associated with Pb. Several of the DNAm changes we identified were in genes linked to neurological function and development. This is especially noteworthy given Pb is a neurotoxicant, and exposure to Pb has been associated with neuronal death, intraneuronal regulatory mechanisms, and neurotransmission, among others (Lidsky and Schneider 2003). Furthermore, it is consistent with previous studies that have found Pb-related changes in neuroepigenetic signaling pathways in human embryonic stem cells (Senut et al. 2014), correlations between WBLLs and DNAm of an important tumor suppressor gene, *p16,* in Pb-exposed individuals (Kovatsi et al. 2010), promoter methylation of *COL1A2* in women (Hanna et al. 2012), and an association between maternal Pb levels and a child’s DNAm profile (Goodrich et al. 2016; Rygiel et al. 2020; Sen et al. 2015). Similar results have been shown in animal models wherein the effects of developmental Pb exposure on the expression of DNA methyltransferases *DNMT1* and *DNMT2,* and the methyl cytosine binding protein *MeCP2* have been identified in rat hipoccampi (Schneider et al. 2013).

Significant DMPs were found in the genes *KDM1A*, *ZMIZ1,* and *SOX18* that have been previously linked to neurological function and development, highlighting the fact that the nervous system is the most affected by Pb exposure. *Lysine demethylase 1A*, or *KDM1A,* is a H3K4 histone demethylase. Mutations in this gene have been linked to neurological conditions (Ricq et al. 2016). *Zinc Finger MIZ-Type Containing 1*, or *ZMIZ1,* is a transcriptional co-activator known to interact with the androgen receptor, and its nucleotide variants have been associated with neurodevelopmental disorders (Carapito et al. 2019; Fewings et al. 2017). *SOX18* (SRY [sex determining region Y] box 18) is a transcription factor involved in the regulation of embryonic development and cell fate determination. When mutated, it can lead to cardiovascular dysfunction (Downes and Koopman 2001; Pennisi et al. 2000). Previous research has shown that downregulation of *Sox18* in male rat hippocampi is associated with postnatal exposure to Pb acetate (Schneider et al. 2012). Using pathway enrichment analysis, we identified the “Neuronal System” pathway, further suggesting that among the top hits in our analysis, genes associated with the neuronal system were statistically overrepresented.

We identified 45 significant DMRs associated with Pb (Sup. Table 3). Most of the DMRs showed decreased methylation as a function of Pb exposure, which is consistent with previous findings (Faulk et al. 2013). According to functional annotation in DAVID, nine of the DMRs (*PMRT1, NNAT, DPP6, STRA6, SNAP23, AGAP2, ZNF385A, and EPHA2*) were associated with neurogenesis, neurological system process, neuron differentiation, and neurodegeneration (Dennis et al. 2003) (Table 3). Seven genes were associated with metal ion binding including *ZNF292*, *ZNF710*, *ZNF385A*, *ZBTB16*, *DPP6*, *AGAP2* and *MEX3A* (Table 3). Interestingly, *NNAT* or neuronatin is an imprinted gene that is paternally expressed and is known to play an important role in brain development (Evans et al. 2001; Joseph et al. 1995). Its methylation levels are decreased in cord blood of newborns whose mothers where exposed to heavy metals during pregnancy (Vidal et al. 2015). Together, these findings are important given that exposure to Pb has been linked with neurodegeneration in adults and neurodevelopmental effects in children (Needleman et al. 1979; Stewart et al. 2006), and further highlight the potential role of DNAm as a marker for environmental exposures on the body.

One way through which Pb could affect DNAm is through ROS, given environmental metals, including Pb, can increase the production of ROS via redox cycling (Adonaylo and Oteiza 1999; Ahamed and Siddiqui 2007). This, in turn, can influence epigenetic processes, and specifically DNAm. Oxidative damage to the DNA can hamper the ability of DNA methyltransferases to interact with the DNA, which then may result in altered DNAm levels (Baccarelli and Bollati 2009).

We found higher WBLLs in individuals recruited in Cerro de Pasco compared to the individuals recruited in Lima, which is consistent with previous studies conducted in Cerro de Pasco. Our findings are similar to Conklin et al. (2008) who reported 5.8 ug/dL mean Pb in women of child-bearing age in Cerro de Pasco (Conklin L. et al. 2008). We observed a positive association between WBLLs measured in residents of Cerro de Pasco and proximity to the Cerro de Pasco mine. Furthermore, WBLLs were associated with interpolated soil Pb values that also tended to be higher close to the mine. These results support what has been reported for the residents of Cerro de Pasco on the relationship between soil Pb measurements of the house and blood Pb levels (Conklin L. et al. 2008).

Interestingly, we identified a positive association between WBLLs and Hb concentration. This finding is at odds with previous studies conducted at low altitude demonstrating elevated WBLLs can lead to decreased Hb levels among adults and children (iron deficiency anemia) (Papanikolaou et al. 2005). This decrease occurs as a result of Pb binding to Hb when circulating in the blood, competing with iron, and impairing heme synthesis (Barbosa et al. 2005; Barltrop and Smith 1972). One possible explanation for the positive association between Pb and Hb at high altitude (4,400 m) is the Andean adaptive response to high altitude (Beall 2007). At altitude, environmental hypoxia triggers erythropoiesis through increased production of erythropoietin (EPO) that stimulates RBC and Hb production allowing for greater oxygen-carrying capacity (Guillemin and Krasnow 1997; Knaupp et al. 1992; Storz and Moriyama 2008). Andeans are known to have elevated Hb levels at high altitude, which counteract the environmental hypoxia and maintain optimal oxygen saturation (Monge et al. 1992; Moore 2001). Similar to low ambient oxygen tension, heavy metals, including Pb, can activate hypoxia signaling pathways (Galanis et al. 2009; Valko et al. 2005). Exposure to Pb can result in hypoxia-like responses, and potentially induce similar responses in the body as exposure to hypobaric hypoxia. Thus, Pb exposure in Andeans may potentially lead to increased Hb production given that Andeans are adapted to produce higher levels of Hb in hypoxic conditions (Beall 2000). This, in turn, can counteract Pb-induced anemia. In our cohort, the relationship between Hb and Pb was strong at both high and low altitude suggesting that the positive relationship between Hb and Pb is independent of high-altitude hypoxia, but potential associated with lead-induced hypoxia. The positive relationship between Hb and Pb likely only exists at low levels of exposure to metals, since high levels of Pb exposure (>30 μg/dL) tended to decrease Hb in Ecuadorian Andeans, although that relationship was not statistically significant (Counter et al. 2001). On the other hand, it is possible that WBLLs we measured are not representative of the relevant Pb exposure (Chuang et al. 2001; Hu et al. 1994), which could explain why we see the positive association between Pb and Hb, in contrast to other studies. Moreover, individuals recruited in Cerro de Pasco have higher Hb levels associated with living at altitude, which could have influenced our ability to correctly measure WBLLs, due to possible interference of high Hb with blood Pb measurement.

## 5. Limitations

DNAm signatures differ by tissue and cell type (Kitamura et al. 2007). In this study, we used DNA extracted from whole blood representing a mixture of DNA-containing cells. Our statistical models of DNAm were adjusted for the principal components based on the blood cell types determined bioinformatically using the method by Houseman et al. (2012). This allowed us to control for the effect of blood cell composition on DNA methylation signatures.

We use comb-p (Pedersen et al. 2012) to identify differentially methylated regions, which has been shown to have high false-positive rates (Kolde et al. 2016; Suderman et al. 2018).

Diet is known to affect DNAm signatures (Zhang et al. 2011). Lima is an urban center and Cerro de Pasco is a remote, Andean town. Any potential differences in diet between participants recruited in Lima and Cerro de Pasco may contribute to the differences in DNAm that we observed. Given our identification of DMPs and DMRs associated with neurological function, we believe that our findings reflect the effect of Pb, a known neurotoxicant, on the DNAm and not the effect of diet.

XRF soil lead values we used in our analyses were collected in 2009 (van Geen et al. 2012), while blood samples were collected during the summer of 2012 and 2013. This may explain why the correlation we see between soil Pb and WBLLs is not significant, but only approaching significance. It is possible that if the XFR values were more recent, we would see a stronger correlation between soil Pb and WBLLs.

Lima and Cerro de Pasco differ by altitude. Lima is at an altitude of 150 masl, and Cerro de Pasco is at 4,338 masl. Our previous work has shown that exposure to high-altitude hypoxia is associated with differences in LINE-1 global DNAm and *EPAS1* methylation in this cohort (Childebayeva et al. 2019) as well as genome-wide DNAm differences (Childebayeva et al., 2020). It is possible that the DNAm differences observed between participants from Lima and Cerro de Pasco were the result of decreased oxygen availability experienced at high altitude and not from the effects of Pb. We expect this effect to be mitigated by the use of WBLLs in our analyses, which diminishes the confounding between Pb exposure and high-altitude hypoxia given that it is a direct measurement of Pb exposure. Furthermore, our research on DNAm differences associated with altitude have identified different DMRs and DMPs than presented here (Childebayeva et al. 2020).

## 6. Conclusions

We tested the effects of Pb exposure in a cohort of adults from two locations in Peru: Lima, the coastal capital, and Cerro de Pasco, a high-altitude mining town. We demonstrated higher WBLLs in participants from Cerro de Pasco compared to the participants from Lima. We showed a positive association between WBLLs and proximity to the Cerro de Pasco mine and interpolated soil Pb. We also found a positive association between Pb and Hb. Lastly, we identified significant associations between WBLLs and DNAm.

To our knowledge, this is among the first studies to report associations between chronic low-level Pb exposure and DNAm in genes associated with neurodevelopment in adults. Collectively, our data indicate that proximity to mining may have effects on human physiology and epigenetics, even when the levels of exposure are low. We suggest conducting future research on Pb exposure in children living in Cerro de Pasco, as well as pregnant women, since these two groups are especially vulnerable to the negative effects of Pb exposure.

## Supplementary Material

Supplementary Table 1-3

Supplementary Figures 1-4

## Figures and Tables

**Table 1:** Participant characteristics.

Phenotype	Lima (n = 148)	Cerro de Pasco (n = 157)	Total (n = 305)	Lima EPIC (n = 59)^#^	Cerro de Pasco EPIC (n = 28)^#^	Total EPIC (n = 87)^#^
Age	24.83 ± 5.05	24.67 ± 4.96	24.75 ± 5.00	24.93 ± 5.14	23.82 ± 4.7	24.57 ± 5
%Female	52%	52%	52%	36%	36%	36%
Hb, g/dL*	13.74 ± 1.40	17.50 ±2.24	15.66 ± 2.66	14.11 ± 1.49	18.03 ± 1.65	15.37 ± 2.4
Height (cm)*!	159.80 ± 8.58	157.59 ± 8.08	158.67 ± 8.39	161.8 ± 7.38	158.93 ± 7.91	160.88 ± 7.63
Weight (kg)*	64.62 ± 11.07	59.46 ±7.93	61.96 ± 9.91	67.1 ± 10.34	60.38 ± 7.52	64.93 ± 9.99
BMI*	25.23 ± 3.43	23.98 ± 3.09	24.59 ± 3.32	25.6 ± 3.41	23.98 ± 3.27	25.08 ± 3.43
Education^^^	NA	2.79 ± 0.41	NA	NA	2.79 ± 0.42	NA
WBLL, µg/dL*	2.03 ± 0.62	4.75 ± 1.53	3.43 ± 1.53	2.07 ± 0.66	5.18 ± 1.70	3.08 ± 1.83

Data and means ± SD

^ 1 = primaria (primary school), 2 = secundaria (secondary school), 3 = Tec/Superior (higher education)

Hb, hemoglobin; BMI, body mass index; WBLL, whole blood lead level

# participant characteristics for individuals analyzed for DNA methylation

* p-value < 0.05 based on linear regression in R for the comparison of Lima versus Cerro de Pasco

! p-value not significant in the subset of individuals analyzed for DNA methylation

**Table 2:** Significant differentially methylated positions (DMPs) associated with WBLLs.

Gene	CpG site	P-value	Q-value	Chr:pos	Coefficient	Beta diff.*	Relation to Island**
							
*KDM1A*	cg22225928	2.80E-07	0.08	chr1:23358458	0.08	3%	Open Sea
*ZMIZ1*	cg20543544	3.67E-07	0.08	chr10:81003657	−0.24	−5%	Island
*TERT*	cg17166338	3.77E-07	0.08	chr5:1295969	0.15	3%	Island
*SOX18*	cg01355392	5.66E-07	0.09	chr20:62679255	−0.13	−6%	N. Shore

* Difference in average Methylation beta values between Cerro de Pasco and Lima

** According to an annotation from Illumina

The model was adjusted for study group, sex, age, and blood principal components

WBLL = Whole blood lead levels

**Table 3:** Significant differentially methylated regions (DMRs) associated with metal ion binding and neurological pathways.

*Gene*	Region	Relation_to_Island*	N probes	P-value region	Corrected p-value	Δ Meth**	Relevant function***
*ZBTB16*	chr11:113933513–113935052	S. Shore	7	1.33E-08	5.65E-06	↑	Metal ion binding
*ZNF710*	chr15:90543224–90544600	N. Shore	6	1.63E-07	7.75E-05	↓	Metal ion binding
*ZNF292*	chr6:87861261–87862482	N. Shore	6	7.16E-07	0.000385	↓	Metal ion binding
*MEX3A*	chr1:156046344–156046833	Island	4	2.05E-08	2.74E-05	↓	Metal ion binding
*DPP6*	chr7:153583318–153584874	Island	13	4.54E-06	0.001913	↑	Neurodegeneration; metal ion binding; metal ion transport
*HOXB3*	chr17:46647787–46653712	Open Sea	21	5.77E-14	6.39E-12	↓	Neurogenesis
*NNAT*	chr20:36148133–36149751	N. Shore	38	2.01E-08	8.15E-06	↓	Neurogenesis; neuron differentiation
*PRMT1*	chr19:50190825–50191684	N. Shore	6	3.98E-07	0.000304	↓	Neurogenesis; neuron differentiation
*EPHA2*	chr1:16472001–16473144	N. Shelf	3	8.32E-07	0.000477	↓	Neurogenesis; neuron differentiation
*STRA6*	chr15:74495109–74496041	OpenSea	9	9.2E-06	0.006455	↓	Neurological system process
*ZNF385A*	chr12:54763081–54764372	Island	6	5.25E-08	2.67E-05	↓	Neurological system process; Metal ion binding
*AGAP2*	chr12:58131681–58133184	Island	10	5.57E-10	2.43E-07	↓	Neuron death; regulation of neuron apoptosis; Metal ion binding
*SNAP23*	chr15:42800679–42800834	Open Sea	2	5.06E-06	0.02117	↑	Neurotransmitter transport; regulation of neurotransmitter levels

* According to annotation from Illumina

** Direction of association with WBLLs

*** According to functional annotation from DAVID
