# Supplementary material for: Blood lead levels in Peruvian adults are associated with proximity to mining and DNA methylation
Source: Environ Int. Author manuscript; Available in PMC 2023 Feb 7. (PMC9903334; doi:10.1016/j.envint.2021.106587)
Supplement: Supplementary Figures 1-4 [file NIHMS1858961-supplement-Supplementary_Figures_1-4.pptx]

## Slide 1
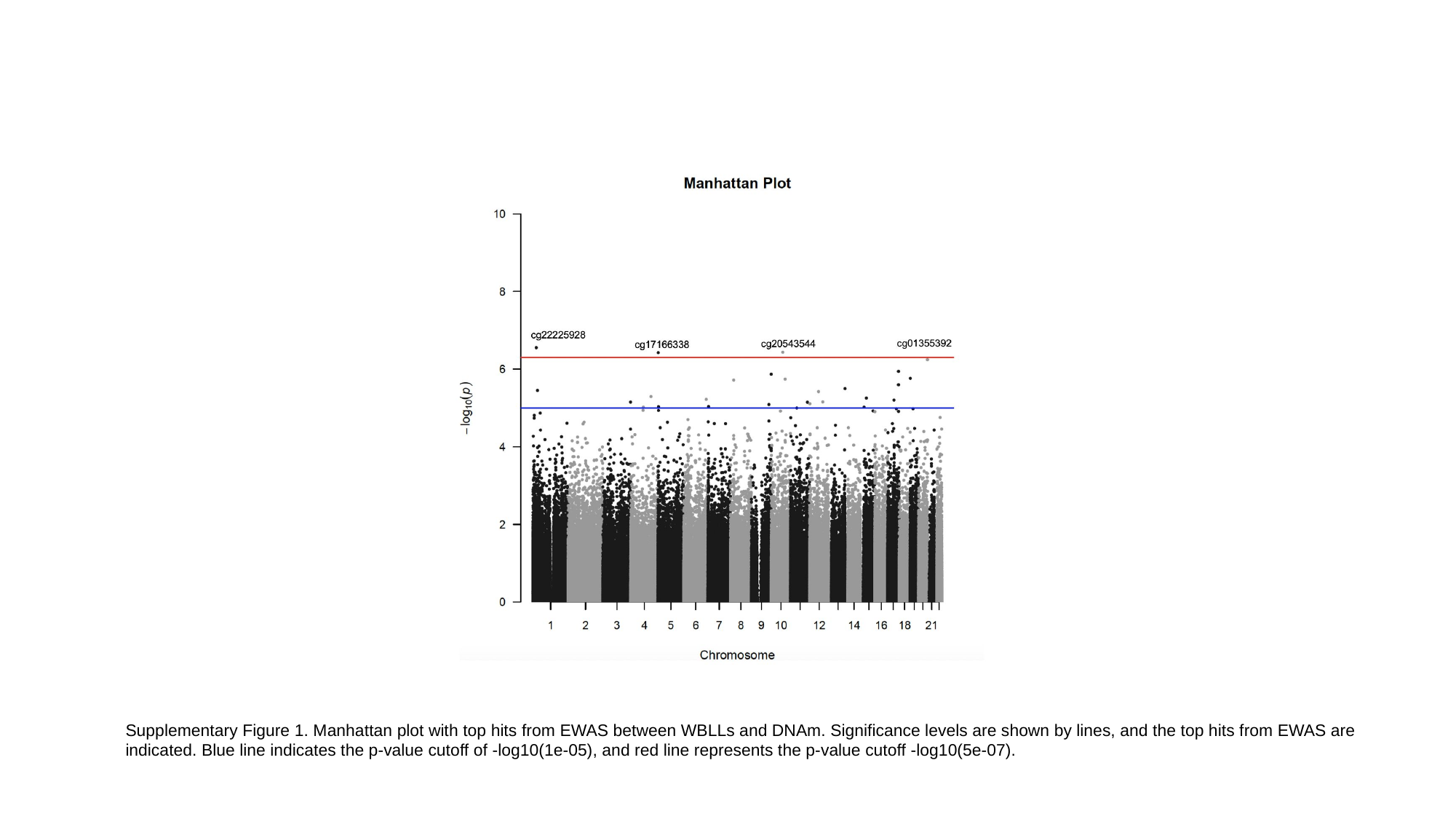

Supplementary Figure 1. Manhattan plot with top hits from EWAS between WBLLs and DNAm. Significance levels are shown by lines, and the top hits from EWAS are indicated. Blue line indicates the p-value cutoff of -log10(1e-05), and red line represents the p-value cutoff -log10(5e-07).

## Slide 2
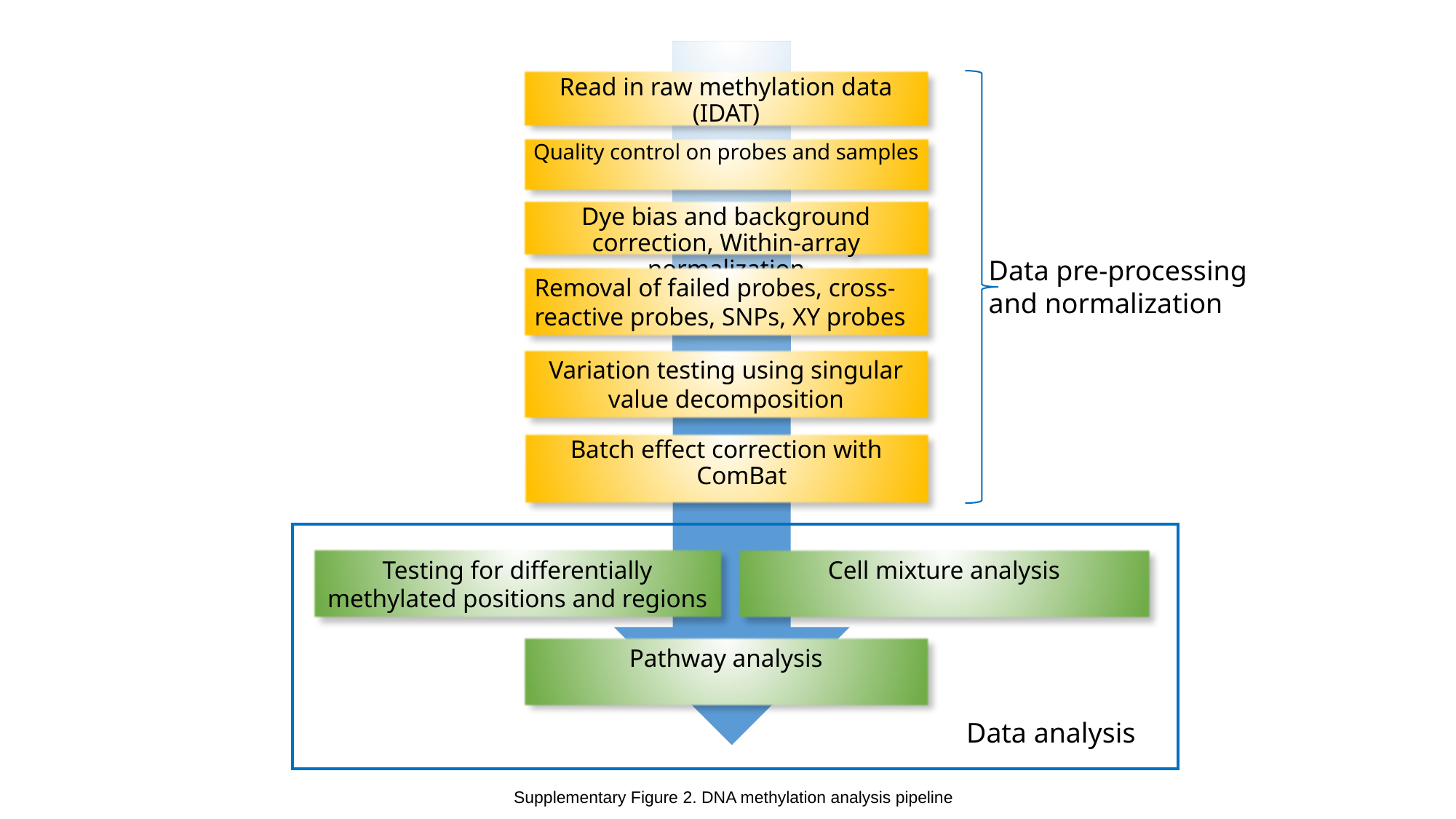

Read in raw methylation data (IDAT)
Quality control on probes and samples
Dye bias and background correction, Within-array normalization
Data pre-processing
and normalization
Removal of failed probes, cross-reactive probes, SNPs, XY probes
Variation testing using singular value decomposition
Batch effect correction with ComBat
Cell mixture analysis
Testing for differentially methylated positions and regions
Pathway analysis
Data analysis
Supplementary Figure 2. DNA methylation analysis pipeline

## Slide 3
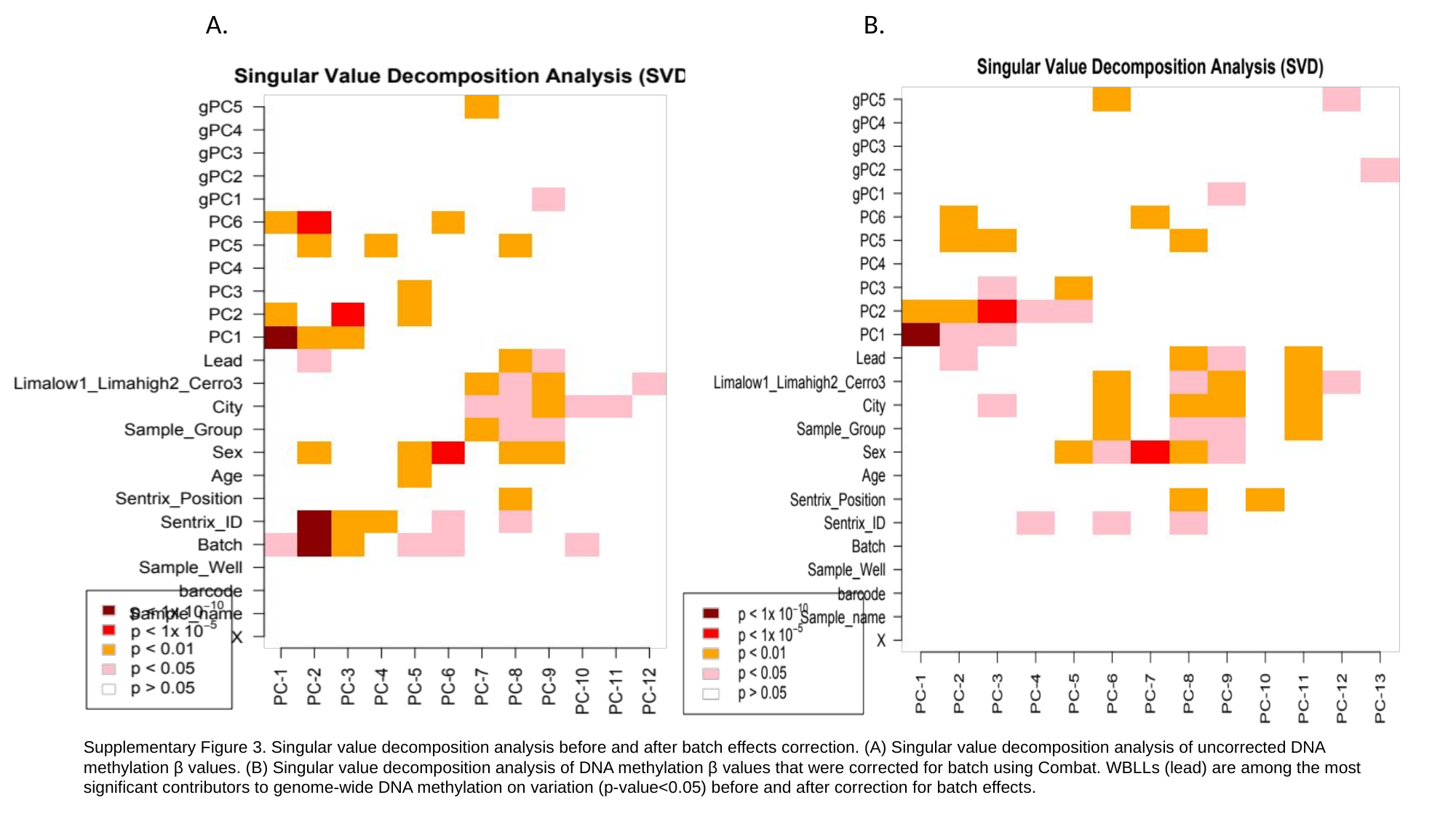

A.
B.
Supplementary Figure 3. Singular value decomposition analysis before and after batch effects correction. (A) Singular value decomposition analysis of uncorrected DNA methylation β values. (B) Singular value decomposition analysis of DNA methylation β values that were corrected for batch using Combat. WBLLs (lead) are among the most significant contributors to genome-wide DNA methylation on variation (p-value<0.05) before and after correction for batch effects.

## Slide 4
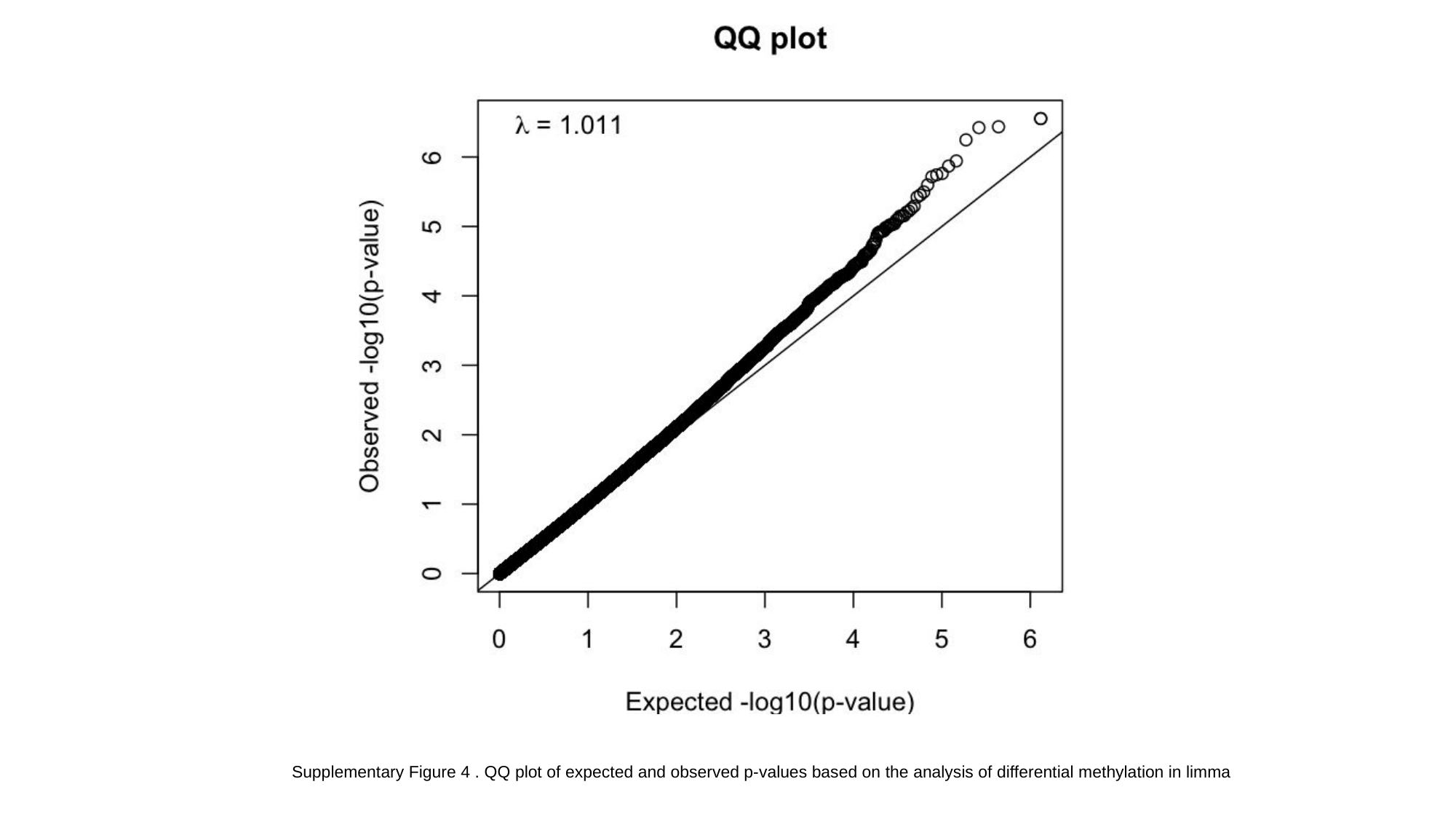

Supplementary Figure 4 . QQ plot of expected and observed p-values based on the analysis of differential methylation in limma
